# Differential immunomodulation in human monocytes versus macrophages by filarial cystatin

**DOI:** 10.1371/journal.pone.0188138

**Published:** 2017-11-15

**Authors:** Gopinath Venugopal, Marion Mueller, Susanne Hartmann, Svenja Steinfelder

**Affiliations:** Institute of Immunology, Centre for Infection Medicine, Freie Universität Berlin, Berlin, Germany; University of North Dakota, UNITED STATES

## Abstract

Parasitic nematodes have evolved powerful immunomodulatory molecules to enable their survival in immunocompetent hosts by subverting immune responses and minimizing pathological processes. One filarial molecule known to counteract host immune responses by inducing IL-10 and regulatory macrophages in mice is filarial cystatin. During a patent filarial infection monocytes encounter microfilariae in the blood, an event that occurs in asymptomatically infected filariasis patients that are immunologically hyporeactive. The microfilarial larval stage was formerly shown to induce human regulatory monocytes and macrophages. Thus, here we aim was to determine how filarial cystatin of the human pathogenic filaria *Brugia malayi* (BmCPI-2) contributes to immune hyporesponsiveness in human monocytes and macrophages elicited by microfilaria. For this purpose, filarial cystatin was depleted from microfilarial lysate (Mf). Detecting the immunomodulatory potential of cystatin-depleted Mf revealed that IL-10, but not IL-8 and IL-6 induction in monocytes and macrophages is dependent on the presence of cystatin. In addition, the Mf-induced expression of the regulatory surface markers PD-L1 and PD-L2 in human monocytes, but not in macrophages, is dependent on cystatin. While Mf-treated monocytes result in decreased CD4^+^ T-cell proliferation in a co-culture assay, stimulation of T-cells with human monocytes treated with cystatin-depleted Mf lead to a restoration of CD4^+^ T-cell proliferation. Moreover, IL-10 induction by cystatin within Mf was dependent on p38 and ERK in macrophages, but independent of the ERK pathway in monocytes. These findings indicate that filarial nematodes differentially trigger and exploit various signaling pathways to induce immunomodulation in different myeloid cell subsets.

## Introduction

Helminths play a central role in causing infections among communities living in tropical and subtropical regions, and about one third of the world population is infected with one or more parasitic helminths[1]. The two major classes of helminths are nematodes and platyhelminths, both of which contain causative agents of a variety of acute debilitating diseases and chronic conditions[2]. In general, helminth infections are well characterized by strong Th2 immune responses, and are able to down-regulate host immune responses[1,3,4].

Lymphatic filariasis (LF) is one of seventeen Neglected Tropical Diseases (NTDs) classified by the World Health Organization (WHO)[5], and is the third leading parasitic cause of morbidity and disability worldwide. LF is normally associated with varied clinical outcomes and according to WHO Global Health Estimates (GHE), in 2015 alone LF contributed close to 2 million Disease Adjusted Life Years (DALYs)[6]. The causative agents of human LF are three species of parasitic nematodes, namely *Wuchereria bancrofti*, *Brugia malayi*, and *Brugia timori*. Over 120 million people around the world are infected with either of these parasites and about 40 million people suffer from disfigurement and incapacitation for work as a result of LF-induced symptoms like lymphedema, elephantiasis or hydrocele[7]. The lifecycle of these filarial worm species comprises of both mosquito (*Aedes*, *Anopheles*, *Culex* or *Mansonia* spp.) and human stages. Infection with the parasite is initiated when an infected mosquito deposits the infective larvae stage on the skin of a human host during a blood meal. The parasite larvae then reach the afferent lymphatic vessels, where they are able to undergo two moulting stages and eventually mature to adult worms. After mating, females start producing microfilariae, which are then able to circulate in the peripheral blood of the human host. During a subsequent blood meal the mosquito vector is therefore able to pick up circulating blood microfilariae, thus initiating the development of the infective L3 stage larvae and completing the transmission cycle of the parasite.

The host-parasite interaction in LF often results in a chronic infection associated with a wide clinical spectrum, characterized by functional dysregulation of both the innate and the adaptive immune responses[8]. Three of the key clinical manifestations of this disease include asymptomatic infection, despite the presence of the larval blood stages (microfilariae) caused by hyporesponsiveness towards the parasite, chronic pathology with hydrocele, or elephantiasis, with or without active infection displaying a Th1/Th17-biased immune response, and endemic normality, where the host is putatively immune against the parasite.

The longevity of helminth parasites in the mammalian host is primarily determined by their capacity to modulate the immune system. Immunomodulatory helminth-derived products such as protease inhibitors[9–14] and homologues of mammalian cytokines and chemokines[15–18] induce a modified Th2-type immune response, which creates a beneficial microenvironment for the parasite[19,20]. Cystatins, a family of cysteine protease inhibitors, are known to regulate cysteine protease functions such as protein catabolism, antigen processing, inflammation, dystrophy and metastasis in viruses, bacteria, protozoa, fungi, plants and mammals [21–24]. Helminth cystatins are highly expressed protease inhibitors and they have been shown to possess strong immunomodulatory properties. They have a dual function, due to their ability to inhibit both parasite and host cysteine proteases. Cystatins are reversible competitive inhibitors of cysteine proteases and control protein processing and turnover, thereby inhibiting antigen processing[10,11], leading to diminished T cell priming. Secondly, they induce the immunosuppressive cytokine IL-10[9] and nitric oxide-producing regulatory macrophages[25], which can result in the inhibition of T cell proliferation, as shown in previous studies on cystatins from the filarial rodent parasites *Acanthoceheilonema viteae* [9] and *Litomosoides sigmodontis* [14], and the human filarial nematode *Onchocerca volvulus* [12].

We could show in previous studies that monocytes and macrophages are target cells of filarial cystatins *in vitro* and *in vivo*[12,13]. Moreover, monocytes from asymptomatic individuals harbouring microfilariae from *Brugia malayi* upregulate programmed-death ligand 1 (PD-L1), IL-10 and IL-8, which is recapitulated in experiments using human monocytes and macrophages from non-endemic healthy donors stimulated with microfilarial lysate (Mf) *in vitro*. Importantly, macrophages differentiated in the presence of Mf lysate respond poorly to LPS, and Mf-stimulated monocytes are able to suppress CD4^+^ T cell functions[26]. This is in line with the finding that patent lymphatic filariasis patients have impaired monocyte and CD4^+^ T cell function, reflected by their inability to produce inflammatory cytokines in response to activating stimuli[27,28], while elevated levels of IL-10 are observed in asymptomatic patients[26,29]. The anti-inflammatory cytokine IL-10 plays a major role in regulating inflammatory diseases such as allergies and autoimmune disorders[30], while IL-8 is an important chemokine with a key role in the pathogenesis of filarial diseases[31] and it displays elevated levels in patients with and without circulating microfilariae[32].

There are three known cystatins from the human filarial nematode *Brugia malayi* (BmCPI-1, -2 and -3). Among these three types, BmCPI-2 is expressed both in the vector and in the mammalian life stages while BmCPI-1 and -3 are exclusively expressed in the mosquito stages of the parasite [10,33]. Most importantly, BmCPI-2 is released in greater amounts by *Brugia malayi* microfilaria compared to adults stages[34]. This prompted us to investigate the immunomodulatory potential of BmCPI-2 on human monocytes and macrophages. Furthermore, we tried to determine the contribution of *Brugia malayi* cystatin in Mf-induced immunomodulation of human monocytes and macrophages, and to further demonstrate the key host signalling events used by BmCPI-2 and Mf to modulate these host cell types. In the present study, we demonstrate that the expression of IL-10 by Mf-stimulated monocytes and macrophages is dependent on cystatins. Moreover, we show that IL-10 expression in monocytes, but not in macrophages, is independent of ERK signalling, suggesting different pathways of IL-10 induction in these cell populations.

## Materials and methods

### Human blood samples

Human blood cells were derived from the German Red Cross and all experiments were approved by the ethics committee of the Charite University, Berlin, Germany.

### Filarial lysate preparation

Live *Brugia malayi* microfilariae were kindly donated by the NIAID/NIH Filariasis Research Reagent Resource Center in Athens, Georgia, USA. The worms were initially washed twice in RPMI medium containing 1% glucose, 200 U/ml penicillin and 200 μg/ml streptomycin, and incubated with the same medium at 37°C and 5% CO_2_ for 24h, followed by washing in phosphate buffered saline (PBS). To prepare the filarial lysate, microfilariae worms were homogenised directly in a glass homogeniser and ultrasonicated on ice at an intensity of 10% for 3 min. The homogenate was centrifuged at 12,000 rpm at 4°C for 10 min and sterile filtered through a 0.22 μm filter. Protein concentration from the filtered homogenate was determined by using the Pierce™ BCA protein assay kit (Thermo Scientific, Rockford, IL, USA), as per the manufacturer’s guidelines.

### Immunoprecipitation of *Brugia malayi* cystatin

An immunoprecipitation technique was performed to deplete *Brugia malayi* cystatin from protein mixtures of microfilarial lysate (Mf) by using rabbit polyclonal antibodies raised against BmCPI-2, recombinantly expressed in *E*. *coli*. The assay was performed using an immunoprecipitation kit from Novex (Thermo Fisher Scientific, Waltham, MA, USA), as per the manufacturer’s guidelines. Briefly, 50 μl of protein G-coupled Dynabeads® were added to the buffer containing anti-BmCPI-2 antibodies (1:1000). After 10 min incubation, the supernatant was removed and beads were washed twice. Then, 100–150 μl of Mf was added to the beads. After 10–15 minutes incubation, the beads were removed, cystatin-depleted Mf was collected and the protein concentration was determined using the Pierce™ BCA protein assay kit (Thermo Scientific, Rockford, IL, USA), as per the manufacturer’s guidelines.

### Cloning of the BmCPI-2 sequence into the LEXSY expression system and purification of BmCPI-2

The nucleotide sequence of BmCPI-2 was amplified from *Brugia malayi* microfilaria cDNA using specific primers (forward: 5’-gTCgACgCTTTgATTCATCgACgAg-3’, reverse: 5‘-ggTACCTACTgACgAgAgTACCTTTg-3‘) including restriction sites for cloning the sequence into the pLEXSY-sat2 plasmid of the LEXSYcon2 Expression Kit (Jena Bioscience GmbH, Jena, Germany). The amplified sequence did not include the coding region for the specific signal peptide of BmCPI-2. Cloning procedures followed the manufacturer’s instructions and resulted in a monoclonal LEXSY cell strain expressing and secreting BmCPI-2 with a C-terminal hexa-histidine tag into the culture medium. Purification of the protein was performed via affinity chromatography using HisTrap™excel columns and the ÄKTA™ pure chromatography system (GE Healthcare Bio-Science AB, Uppsala, Sweden) with a non-denaturing protocol, with imidazole as a competitive eluent. The purified protein was dialysed against PBS and sterile filtered. The protein concentration was determined using the Pierce™ BCA Protein Assay Kit (Pierce Biotechnology, Rockford, USA).

### Immunoblot analysis

SDS-PAGE was performed in 12% separating gel with 6% stacking gel. 15 μg of protein from each sample was applied per lane. Proteins were transferred to a nitrocellulose membrane for immunodetection. After blocking with 5% non-fat dry milk in TBS-Tween, membranes were incubated with rabbit anti-BmCPI-2 (1:50,000), followed by incubation with horseradish peroxidase conjugated anti-rabbit IgG (1:15,000) (Cell signalling, Danvers, MA, USA). Signals were detected by a chemiluminescence reaction using the ECL kit (Amersham, GE Healthcare Europe, GmBH). All bands were visualized by an enhanced chemiluminscence system (PEQLAB, Erlangen, Germany).

### Protein detection by silver staining

Silver staining was performed to detect proteins separated in SDS-PAGE by using Thermo Scientific Pierce Silver Stain kit (Thermo scientific, Rockford, IL, USA) using the manufacturer’s protocol. Briefly, 15 μg of protein from each sample was applied per lane in SDS-PAGE. After running the gel for 1 h, the gel was washed twice with ultrapure water and then fixed twice by adding 30% ethanol and 10% acetic acid for 15 min each. After 30 min of fixation, the gel was washed twice, first with 10% ethanol then with ultrapure water. After the washing step, the gel was sensitized quickly for 1 min using silver stain sensitizer then washed twice with ultrapure water. The gel was then incubated for 30 min with a silver stain enhancer and washed briefly with ultrapure water. The gel was developed using silver stain developer solution and the development procedure was stopped immediately after the desired band intensity was achieved using 5% acetic acid. After the addition of stop solution, the gel was washed briefly with ultrapure water and incubated for 10 min at room temperature.

### Isolation of CD14^+^ monocytes and differentiation protocol for monocyte-derived macrophages from human PBMCs

Peripheral blood mononuclear cells (PBMCs) were isolated from buffy coats by density gradient centrifugation using Pancoll human (PAN Biotech, Aidenbach, Bayern, Germany). To positively select for CD14^+^ monocytes, 200 μl of anti-CD14 beads (Miltenyi Biotec, Bergisch-Gladbach, Germany) were added to 5-10x10^8^ PBMCs for 20 min and cells were separated by an autoMACS classic (Miltenyi Biotec, Bergisch-Gladbach, Germany) using the program ‘Possel’. Macrophages were generated *in vitro* by culturing CD14^+^ monocytes in complete RPMI containing 10 ng/ml human M-CSF in 6-well cell culture plates at a cell concentration of 0.33 x 10^6^/ml and a density of 0.1x10^6^/cm^2^ for 6 days, at 37°C and 5% CO_2._

### Cell culture and stimulation of monocytes and macrophages

The CD14^+^ monocytes and monocyte-derived macrophages were stimulated with parasite material or recombinant BmCPI-2 and kept for 24 h at 37°C and 5% CO_2_. The supernatant was collected after 24 h and stored at -20°C for further analysis. In experiments including inhibitors, the inhibitors were added to the cell culture 60 min before stimulation. The following inhibitors were used in our study: p38 inhibitor (SB203580), and MEK1/2 inhibitor (U0126) (both from Calbiochem, Merck, Darmstadt, Germany).

### Flow cytometry

Monocytes and macrophages were analysed for surface expression of PD-L1 and PD-L2. Cells were treated with FcR blocking reagent (Miltenyi Biotec, Bergisch-Gladbach, Germany), stained with fixable viability dye eFluor 780 (eBiosciences, San Diego, USA) and additionally stained for the following markers: anti-274-PE (clone MIH1), anti-273-PE (clone MIH18). Fixed cells were acquired using the FACS Canto II (BD, Franklin Lakes, USA) and analysed using FlowJo, version 10.2 (Tree Star, Ashland USA).

### Co-culture of CD4^+^ T cells and monocytes

To perform the co-culture experiments for a functional assay of T cell proliferation, CD4^+^ T cells were isolated from PBMCs using the CD4^+^ T cell microbeads (Miltenyi Biotec, Bergisch-Gladbach, Germany) according to the manufacturer’s instructions and sorted on an autoMACS classic (Miltenyi Biotec, Bergisch-Gladbach, Germany) using the program ‘Possel’. The autologous CD4^+^ T cells were stained for CFSE and rested in complete RPMI for 24 h at 37^o^ C and 5% CO_2_.

Monocytes were left unstimulated or stimulated with 20 μg/ml of Mf or cystatin-depleted Mf lysate for 24 h in a 5% CO_2_ incubator at 37^o^ C in 24 well plates. 5x10^5^ CFSE-labelled CD4^+^ T cells were co-cultured with 1x10^5^ stimulated monocytes in 96 well flat bottom plates in the presence of 2 μg/ml soluble anti-CD3 (OKT3, eBioscience, San Diego, USA). After 5 days, the cells were washed and stained with fixable viability dye eFluor 780 (eBioscience, San Diego, USA) and anti-CD4-PerCP (clone RPA-T4, BioLegend, San Diego, USA). Fixed cells were acquired using the FACS Canto II (BD, Franklin Lakes, USA) and analysed using FlowJo, version 10.2 (Tree Star, Ashland USA).

### Cytokine detection by ELISA

IL-10, IL-8, IL-6 and TNFα proteins were measured using commercial enzyme-linked immunosorbent assay (ELISA) kits (eBioscience, San Diego, CA, USA). IL-12p40 was also measured using an ELISA kit (BioLegend, San Diego, CA, USA). All samples were measured in duplicates. Absorbance was read at 450nm with background wavelength subtracted at 570nm using a Synergy HT plate reader (BioTek, Winooski, VT, USA).

### Statistical analysis

All statistical analyses were performed using GraphPad Prism version 7.0 (GraphPad Software, Inc., CA, USA). Comparisons between groups were performed using the Wilcoxon signed rank test.

## Results

### BmCPI-2 from *Brugia malayi* induces IL-10, IL-8, IL-6 and PD-L1/L2

**Fig 1:**
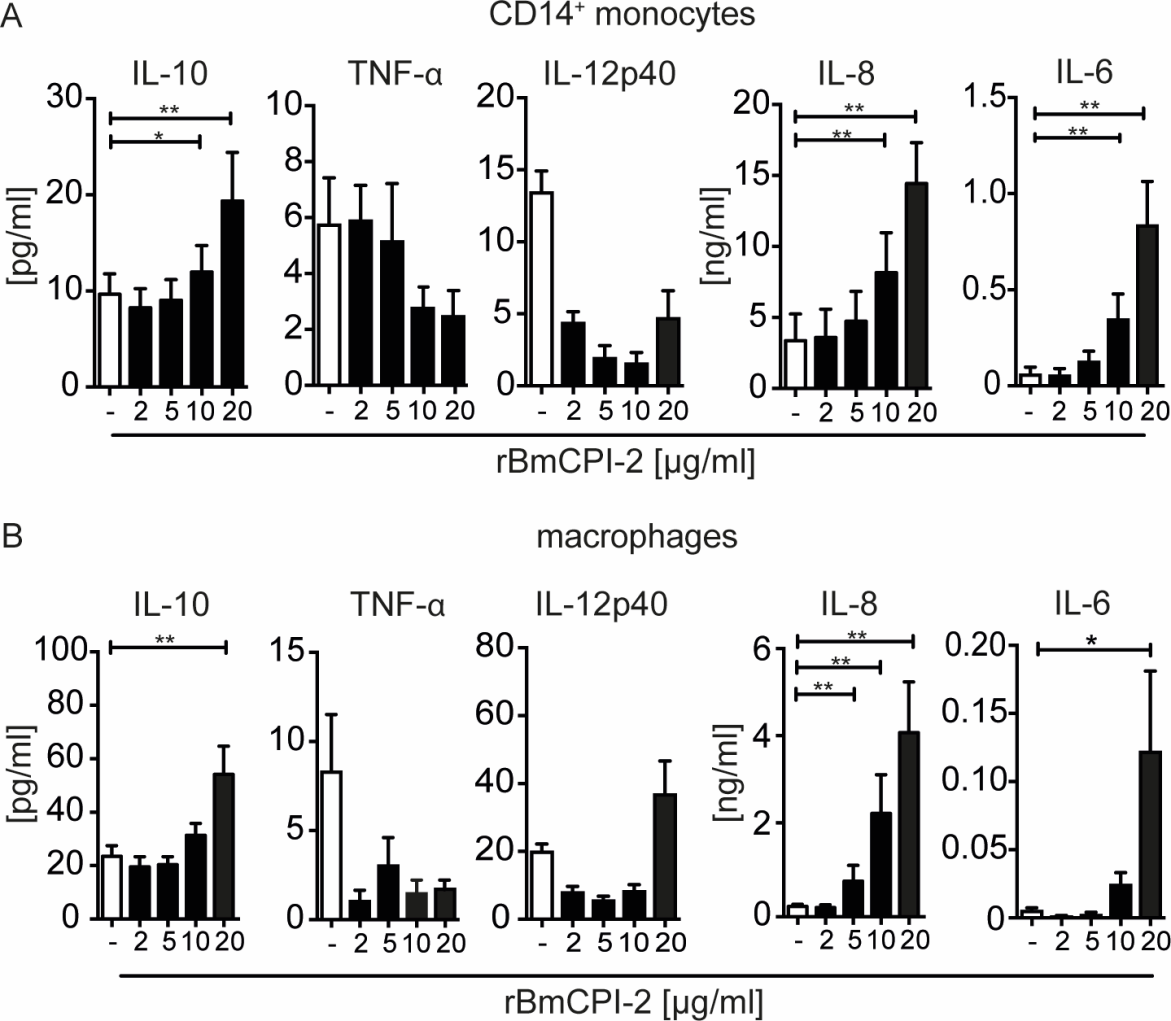
*Brugia malayi* cystatin-2 (rBmCPI-2) induces IL-10, IL-8 and IL-6 in human monocytes and macrophages. Human monocytes (A) and macrophages (B) were stimulated with different concentrations [μg/ml] of recombinant BmCPI-2 for 24h. Cytokines in the supernatant were detected by ELISA. Data are from 12 donors and shown as mean ±SEM. Statistical analysis was done using the Wilcoxon matched-pairs signed rank test in reference to the unstimulated control. *p<0.05, **p<0.01.

**Fig 2:**
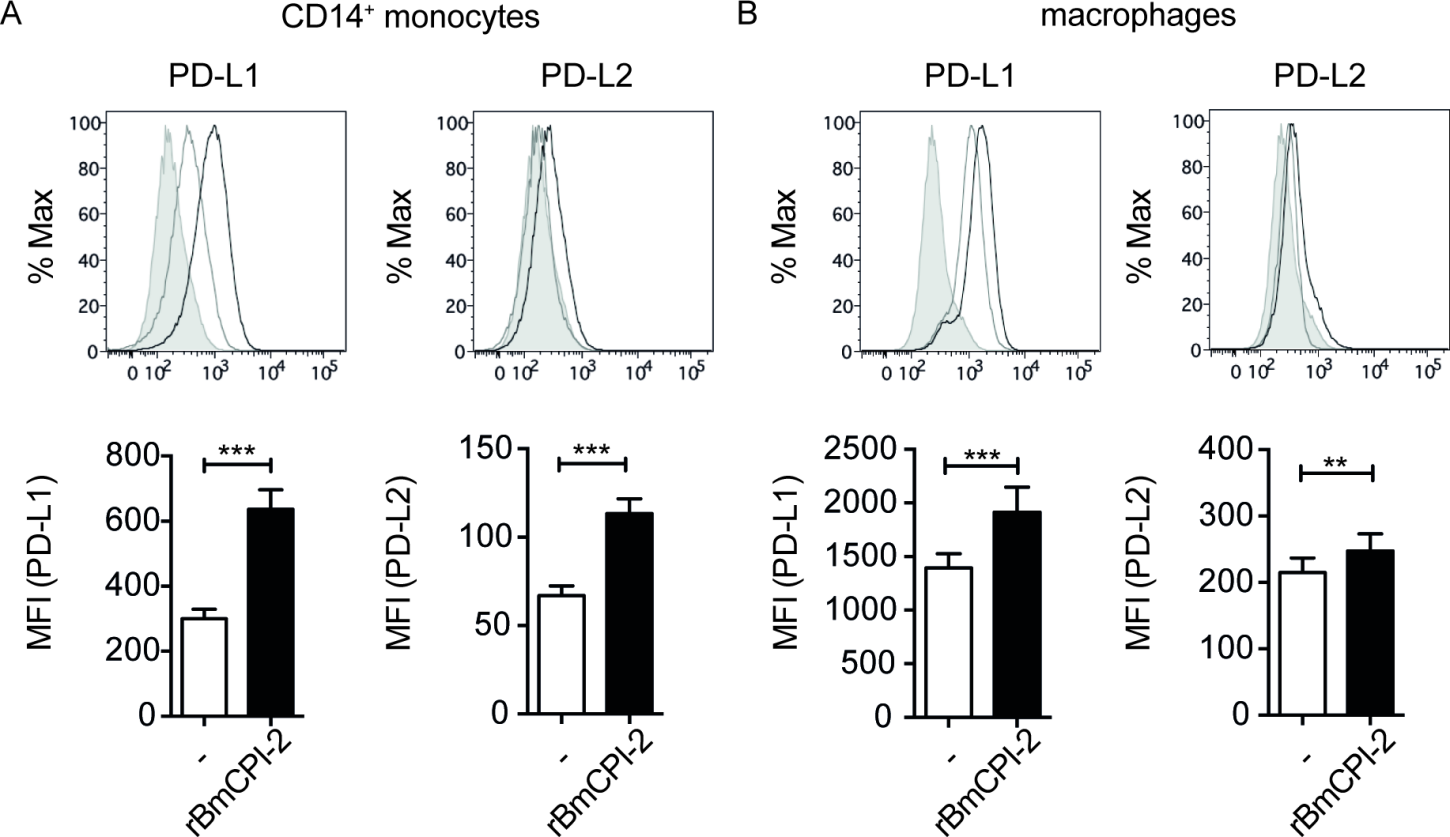
rBmCPI-2 induces expression of PD-L1 and PD-L2 on human monocytes and macrophages. Monocytes (A) or macrophages (B) were left unstimulated or stimulated for 24 h with 20 μg/ml of recombinant BmCPI-2 (n = 12). Surface expressions of PD-L1 and PD-L2 were measured by flow cytometry. Histograms in the upper panel show representative plots of unstained (grey tinted), unstimulated (grey line) and stimulated cells (black line). Bar graphs in the lower panel show the mean fluorescence intensity (MFI). All data are represented as mean ±SEM. P values were calculated using Wilcoxon signed-rank test. **p<0.01, ***p<0.001.

We have previously shown that *Brugia malayi* microfilarial lysate (Mf) induces a regulatory subset of human monocytes expressing IL-10 and PD-L1 leading to inhibition of CD4^+^ T-cell proliferation and cytokine secretion[26]. In the present study, we hypothesized that the observed immunomodulation seen might be due to the presence of *Brugia malayi* cystatin, as the immunomodulatory potential of cystatin has been demonstrated in murine macrophages[9,14]. First, to determine the potential of BmCPI-2 to induce a regulatory phenotype in human monocytes and macrophages, we tested if BmCPI-2 is able to induce inflammatory and anti-inflammatory cytokines and programmed death-ligands in human monocytes and macrophages of healthy blood donors in a similar fashion as Mf[26]. To this end, we used recombinant BmCPI-2 (rBmCPI-2) that was expressed in a eukaryotic protein expression system. rBmCPI-2 stimulated monocytes to secrete IL-10, IL-8 and IL-6 in a dose-dependent manner, while IL-12p40 and TNFα were not induced (**Fig 1A)**. Likewise, rBmCPI-2 induced IL-10, IL-8 and IL-6 in a similar fashion in human macrophages (**Fig 1B**). The observed increase in IL-12p40 production in human macrophages was not significant between unstimulated cells and cells stimulated with 20 μg/ml of rBmCPI-2 (Fig 1B). Moreover, rBmCPI-2-stimulated monocytes and macrophages showed significant up-regulation of PD-L1 and PD-L2 (**Fig 2**).

### Induction of IL-10 and PD-L1 and PD-L2 in human monocytes by *Brugia malayi* microfilarial lysate is dependent on cystatins

**Fig 3:**
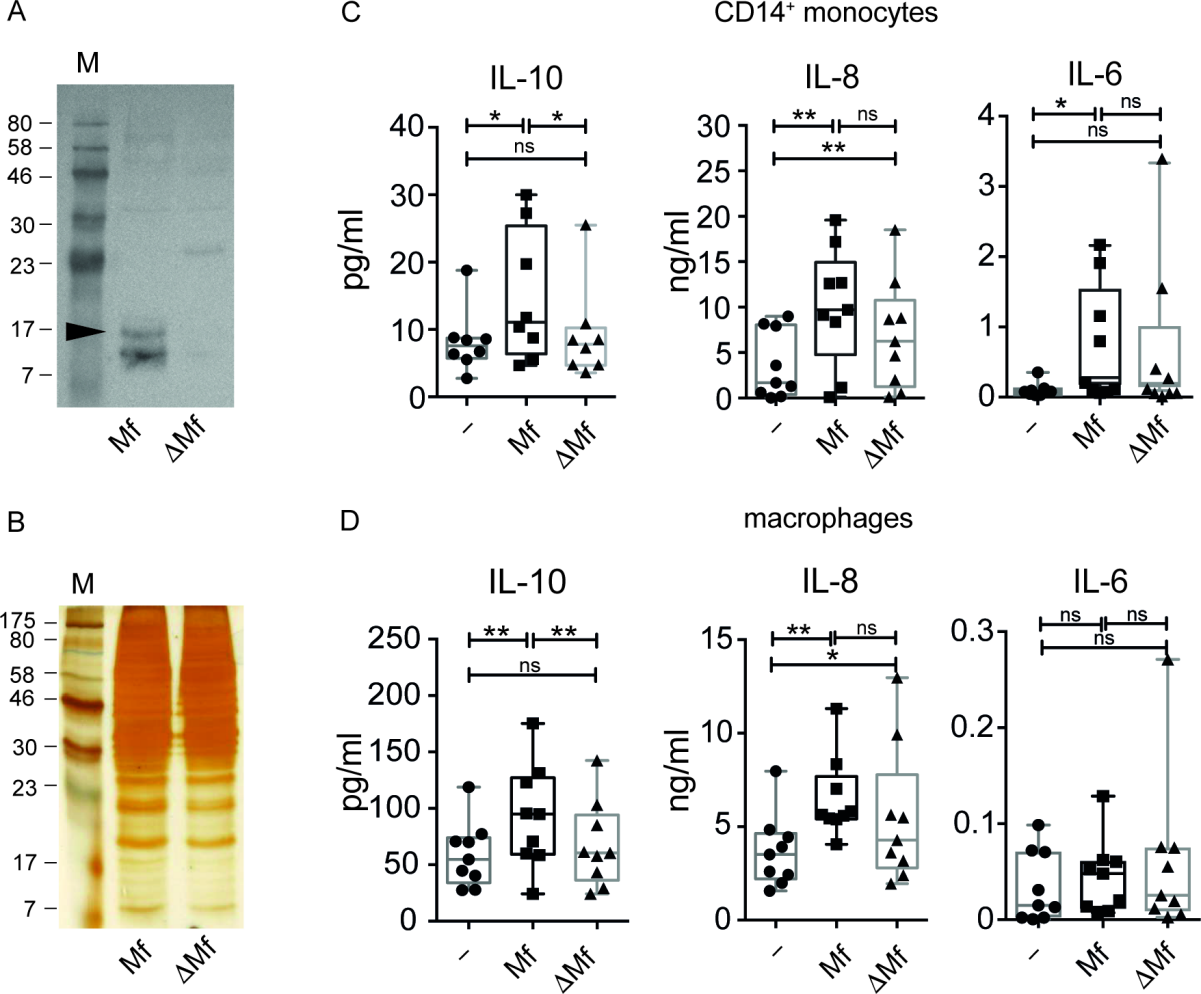
*Brugia malayi* microfilaria lysate induced IL-10 is dependent on the presence of filarial cystatins. Western Blot (A) and Silver gel (B) showing *Brugia malayi* microfilaria lysate (Mf) and microfilaria lysate depleted of filarial cystatins (ΔMf). The black arrow indicates BmCPI-2. C) Human monocytes, and D) monocyte-derived macrophages were stimulated with 20 μg/ml of Mf or ΔMf for 24 h. Cytokines in the supernatant were detected by ELISA. Data are from 8–9 donors and shown as mean ±SEM. Statistical analysis was done using the Wilcoxon matched-pairs signed rank test. *p<0.05, **p<0.01.

To determine if IL-10 production by microfilarial lysate (Mf) in human monocytes and macrophages is dependent on the presence of filarial cystatin, we depleted all three forms of cystatins from Mf (termed here ΔMf) by immunoprecipitation using polyclonal antibodies raised against recombinant BmCPI-2. BmCPI-2 was indicated by a black arrow in Fig 3A. Successful depletion of *Brugia malayi* cystatins from Mf was obtained without altering the overall protein composition of Mf (**Fig 3A and 3B**). Monocytes and macrophages stimulated with ΔMf did no longer produce significant amounts of IL-10, similar to unstimulated controls, in contrast to non-cystatin-depleted-Mf-stimulated monocytes (**Fig 3C)** and macrophages (**Fig 3D**), which induced significant amounts of IL-10. These data suggest that IL-10 induction by Mf is strictly cystatin-dependent in monocytes and macrophages.

**Fig 4:**
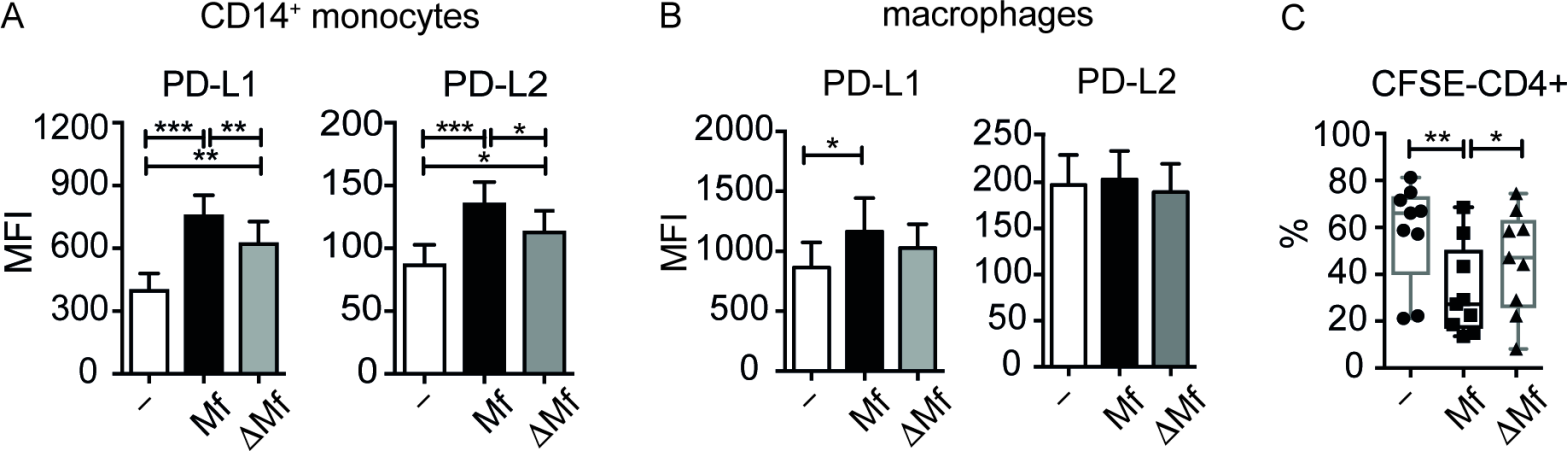
Mf-induced PD-L1 and PD-L2 on monocytes is dependent on the presence of filarial cystatins. A) Human monocytes, and B) monocyte-derived macrophages were stimulated with 20 μg/ml of Mf or ΔMf for 24 h. Surface expression of PD-L1 and PD-L2 was measured by flow cytometry. Bar graphs show the mean fluorescence intensity (MFI). Data are from 16 (monocytes) and 9 (macrophages) donors, respectively, and shown as mean ±SEM. C) Flow cytometric analysis of CD4^+^ T cells. 5x10^5^ CFSE-labelled CD4^+^ T cells were co-cultured for 5 days with 1x10^5^ monocytes left either unstimulated or stimulated for 24 h with 20 μg/ml Mf or 20 μg/ml ΔMf. Graph shows the percentage of CD4+ T cells that divided. Data are from 9 donors and shown as mean ±SEM. Statistical analysis was done using the Wilcoxon matched-pairs signed rank test. *p<0.05, **p<0.01, ***p<0.001.

On the other hand, Mf-induced IL-8 was not strictly dependent on cystatins, since IL-8 levels were lower but not significantly different in ΔMf- versus Mf-stimulated monocytes and macrophages. Similarly, Mf-induced IL-6 in monocytes was not dependent on cystatins, whereas in macrophages Mf did not induce IL-6 (Fig 3C and 3D). Notably, our results show that Mf-induced expression of PD-L1 and PD-L2 in monocytes is partially dependent on cystatins (**Fig 4A**). On the other hand, expression of PD-L1 in human macrophages was not dependent on cystatins (**Fig 4B**). Next, we employed a functional assay for CD4^+^ T-cell proliferation using autologous monocytes treated with Mf or ΔMf and co-cultured both cell types with anti-CD3. We observed a significant reduction of proliferation when T-cells were co-cultured with Mf-treated monocytes compared to unstimulated monocytes as described before[26]. Strikingly, when T-cells were co-cultured with ΔMf-stimulated monocytes proliferation was not significantly altered with respect to co-culture with unstimulated monocytes. Moreover, ΔMf-stimulated monocytes significantly restored T-cell proliferation compared to Mf-stimulated monocytes (Fig 4C).

Hence, IL-10 induction by Mf, but not IL-8 or IL-6, appears dependent on cystatins in both myeloid cell types. Furthermore, PD-L1 and PD-L2 expression of monocytes but not macrophages is dependent on cystatins. Most importantly, cystatins mediate the inhibitory potential of monocytes, which leads to reduced CD4^+^ T-cell proliferation.

### Differential induction of IL-10 by rBmCPI-2-stimulated myeloid cell population

Previous studies indicate that the increased production of IL-10 in murine macrophages by cystatin from the rodent filarial nematode *Acanthocheilonema viteae* was critically dependent on two mitogen-activated protein kinases, ERK and p38[35]. Nevertheless, the immune signalling events exploited by human filarial cystatin to induce IL-10 in human monocytes and macrophages is still elusive. This directed us to investigate the signalling events activated or altered by rBmCPI-2 and Mf, and leading to IL-10 and IL-8 production in human monocytes and macrophages. To this end, we used various concentrations of inhibitor molecules known to be involved in the regulation of IL-10 production[35].

**Fig 5:**
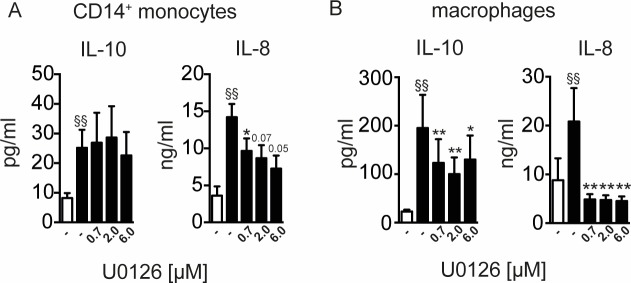
Induction of IL-10, but not IL-8 by rBmCPI-2 in human monocytes is independent of ERK. Human monocytes (A) and macrophages (B) were stimulated with 20 μg/ml of rBmCPI-2 for 24 h with various concentrations of MEK1/2 inhibitor. Cytokines in the supernatants were detected by ELISA. Data are from 7–9 donors and shown as mean ±SEM. Statistical analysis was done using the Wilcoxon matched-pairs signed rank test. Values statistically different from the unstimulated control are depicted as ^§^ p<0.05 and ^§§^ p<0.01. Values statistically different from rBmCPI-2-stimulated monocytes are depicted as * p<0.05 and **p<0.01.

Firstly, we determined the role of the MEK-dependent signalling pathway to induce IL-10 and IL-8 in rBmCPI-2-stimulated human monocytes and macrophages. Monocytes (**Fig 5A**) and macrophages (**Fig 5B**) were incubated with rBmCPI-2 for 24 h in the presence of various concentrations U0126, which is a specific inhibitor of MAP kinase kinases, MEK1 and MEK2, and thus ERK activation. Notably, rBmCPI-2-induced-IL-10 production was independent of ERK signalling, as MEK inhibition did not alter the production of IL-10 in human monocytes (**Fig 5A**), a finding also seen with Mf-stimulated monocytes (**S1 Fig**). In contrast, rBmCPI-2-induced IL-10 production in macrophages was dependent on ERK signalling, as we have observed a significant reduction of IL-10 levels in MEK inhibition (**Fig 5B**). Moreover, we observed a significant dose-dependent reduction of IL-8 levels in rBmCPI-2 (**Fig 5A**) stimulated monocytes and macrophages in the presence of the ERK inhibitor MEK1/2. This finding suggests that IL-10 induction by rBmCPI-2 and Mf is differentially regulated in human monocytes and macrophages.

### Induction of IL-8 and IL-10 by rBmCPI-2-stimulated myeloid cell population is dependent on p38

**Fig 6:**
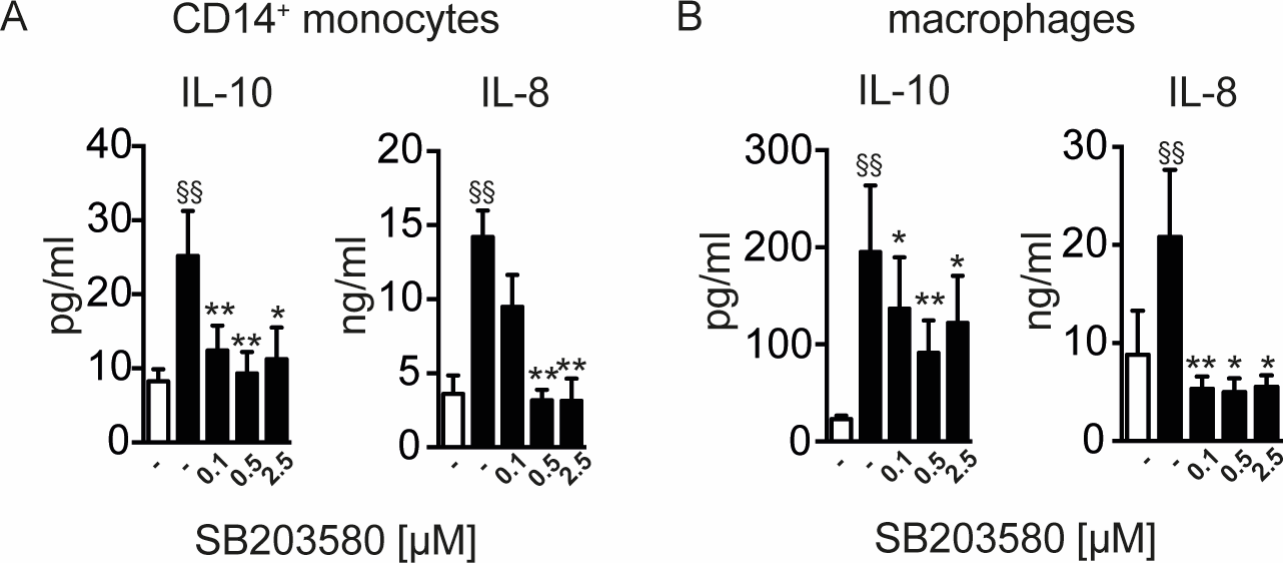
Induction of IL-10 and IL-8 by rBmCPI-2 in human monocytes and macrophages is dependent on p38. Human monocytes (A) and macrophages (B) were stimulated with 20 μg/ml of rBmCPI-2 for 24 h with various concentrations of p38 inhibitor. Cytokines in the supernatants were detected by ELISA. Data are from 7–9 donors and shown as mean ±SEM. Statistical analysis was done using the Wilcoxon matched-pairs signed rank test. Values statistically different from the unstimulated control are depicted as ^§^ p<0.05 and ^§§^ p<0.01. Values statistically different from BmCPI-2-stimulated monocytes are depicted as * p<0.05 and **p<0.01.

We next investigated the possible involvement of the p38-signalling pathway triggered by rBmCPI-2 in inducing IL-10 and IL-8 production in both human monocytes and macrophages. Inhibition of signalling pathways dependent on p38 by addition of the inhibitor SB203580 resulted in a significant reduction of IL-10 and IL-8 in both rBmCPI-2 and Mf-stimulated monocytes and macrophages (**Fig 6A and 6B, S2 Fig**).

## Discussion

The contribution of helminth-derived products towards the modulation of host immune responses has been addressed by several studies, and cystatin is one such secreted protein of filarial nematodes with strong immunomodulatory capacities[19,36]. With the present study we aimed to show the ability of *Brugia malayi* filarial cystatin to induce human monocytes and macrophages with a regulatory phenotype and function.

First, we demonstrated that the nematode immunomodulatory protein BmCPI-2 can significantly induce IL-10, IL-8 and IL-6, but not TNFα and IL-12p40 in human monocytes (**Fig 1A)** and macrophages (**Fig 1B**). Additionally, BmCPI-2 induced the expression of PD-L1 and PD-L2 on the surface of human monocytes and macrophages, suggesting a regulatory monocyte and macrophage phenotype *in vitro* (**Fig 2**). This is in line with our published data on human monocytes and macrophages stimulated with whole lysate of *Brugia malayi* microfilaria (Mf), where monocytes and macrophages develop a characteristic regulatory phenotype with high expression of IL-10 and PD-L1/L2, and inhibit CD4^+^ T cell function[26]. Therefore, we then tested whether the observed immunomodulation seen in human monocytes and macrophages by Mf is due to the presence of cystatin. To that end we analysed the modulatory capacity of Mf that was depleted of cystatin (ΔMf). Interestingly, using polyclonal serum raised against recombinantly expressed BmCPI-2, the protocol resulted in the depletion of all cystatins formerly detectable in Mf. Our results show that Mf-induced IL-10, but not IL-8 and IL-6 production in monocytes and macrophages is due to the presence of cystatin (**Fig 3C and 3D**). This is in agreement with previous studies on the ability of filarial cystatins to induce IL-10 production in murine macrophages[9,37] and human monocytes[12]. Additionally, expression of PD-L1 and PD-L2 in monocytes but not in macrophages is dependent on the presence of cystatin in Mf. Finally, both, the reduced expression of IL-10 and PD-L1/2 in monocytes, which is observed in Mf depleted of cystatins, might contribute to the observed restoration of CD4^+^ T-cell proliferation **(Fig 4)**.

Recently, many studies have been published addressing the therapeutic potential of cystatin in inflammatory diseases due to its anti-inflammatory activity. The modulation of innate immune cells and the profound suppression of the host immune system due to a characteristic anti-inflammatory milieu with increased levels of IL-10 and transforming growth factor (TGF)-β in chronic helminth infections can protect against allergic diseases[38–40]. The importance of IL-10 and of macrophages in suppressing the allergic effects has been described in a murine model of ovalbumin-induced allergic airway hyper-reactivity and DSS-induced colitis applying treatment with filarial cystatin[13]. Moreover, filarial cystatin reduces Respiratory Syncytial Virus (RSV)-inducing immunopathology by inducing high levels of IL-10 production by CD4^+^ T cells in the airways and lungs of mice[41]. Cystatin from the nematode parasite *Ascaris lumbricoides* reduces inflammation in a mouse model of DSS-induced colitis by increasing the expression of IL-10, TGFβ with simultaneous reduction of IL-6 and TNFα[42]. Collectively, the studies described here indicate that cystatins from various nematodes not only function as immunomodulators in the infection setting but also have potential as therapeutic agents for inflammatory diseases. Our results highlight the unique role of cystatin to induce IL-10 in human monocytes and macrophages stimulated with microfilarial products. Also cystatin-dependent expression of PD-L1 and PD-L2 in human monocytes suggests that cystatin from microfilariae is partially involved in the immune regulation process and thus could contribute to asymptomatic infection through PD-L1:PD-1 dependent suppression of CD4^+^ T cell function[26,43–45]. Since circulating microfilariae in asymptomatically infected individuals will readily come into contact with blood monocytes, the modulation of this particular immune cell is vital for the parasite’s immune evasion strategy.

Similar to our previously published results on Mf [26], stimulation of monocytes and macrophages with whole Mf resulted in the production of significantly higher levels of IL-8 compared to unstimulated controls. The significant elevation of IL-8 by Mf-stimulated human monocytes and macrophages, however, was not dependent on cystatin (**Fig 3C and 3D**). Thus, the effect by Mf could be attributed to the presence of other molecules in Mf that are also able to induce IL-8. So far there are only a few studies revealing the potential of filarial products to induce IL-8, such as lipoproteins from *Wolbachia*, an obligate intracellular symbiont of *Brugia malayi*, which was shown to induce IL-8 in a TLR2/6-dependent manner[46]. While infective L3 larvae are not able to induce IL-8 in dendritic cells (DC) [47] or epithelial cells[48], it is expressed in a co-culture of DC and keratinocytes[49]. In contrast to infective larvae, live Mf and excretory/secretory products of female worms can induce the expression of IL-8 in human DC[50] and monocytes[51]. Previously, and also in the present study, we could show significant elevation of IL-6 by Mf in monocytes only, but not in macrophages. Although we see an increased production of IL-6 in Mf-treated monocytes (Fig 3C), IL-6 was not significantly reduced in monocytes that were treated with ΔMf. This result suggests that other molecules from whole Mf lysate were responsible for the elevated levels of IL-6 by monocytes.

Furthermore, we studied the immune signalling events exploited by rBmCPI-2 leading to the production of IL-10 and IL-8 in human monocytes and macrophages. It has previously been demonstrated that ES-62, an excretory-secretory product of the rodent filarial nematode *Acanthocheilonema viteae* induces tyrosine phosphorylation of glycoproteins in murine macrophages[52] and modulates the activation of MAP kinases (ERK, p38 and JNK), thereby regulating cytokine production[53,54]. A study from our own group revealed that IL-10 production in murine macrophages triggered by cystatin from *A*. *viteae* was tyrosine kinase sensitive, and depended on activation of both MAPK[35]. In addition, LPS-stimulated microglia of mice *in vitro* showed that increased IL-10 mRNA expressions by filarial cystatin was dependent on ERK signalling[55]. In agreement with this data, our results indicate a significant reduction of rBmCPI-2- or Mf-induced IL-10 in macrophages, suggesting roles for ERK and p38 in these cells. On the other hand, IL-10 induction by rBmCPI-2 or Mf in human monocytes was independent of ERK signalling, indicating a differential induction of IL-10 in this myeloid cell population. This suggests that filarial products are able to differentially exploit and/or trigger the signalling events in human monocytes and macrophages, leading to induction of IL-10. Moreover, while IL-10 was independent of the ERK pathway in human monocytes only, IL-8 production was dependent on both MAPK kinases (ERK and p38) in monocytes (**Figs 5A and 6A**) and macrophages (**Figs 5B and 6B**).

This is the first study addressing the contribution of filarial cystatin within microfilariae, which circulate in the blood, to induce IL-10 in monocytes. Overall, our results show that IL-10 and IL-8 induction by human filarial cystatin in human monocytes and macrophages is dependent on p38, while only IL-10 induction in macrophages is additionally dependent of ERK signalling. Moreover, Mf-induced IL-10 production, but not IL-8 or IL-6 induction in human monocytes and macrophages is dependent on cystatin, suggesting that while cystatin is sufficient and necessary for IL-10 induction, factors other than cystatin are responsible for IL-8 and IL-6.

## Supporting information

S1 Fig Induction of IL-10, but not IL-8 by microfilarial lysate (Mf) in human monocytes is independent of ERK. Human monocytes (A) and macrophages (B) were left unstimulated or stimulated with 20 μg/ml of Mf for 24 h with various concentrations of MEK1/2 inhibitor. Cytokines in the supernatants were detected by ELISA. Data are from 7–9 donors and shown as mean ±SEM. Statistical analysis was done using the Wilcoxon matched-pairs signed rank test. Values statistically different from the unstimulated control are depicted as ^§^ p<0.05 and ^§§^ p<0.01. Values statistically different from Mf-stimulated monocytes are depicted as * p<0.05 and **p<0.01.
(TIF)

S2 Fig Induction of IL-10 and IL-8 by Mf in human monocytes and macrophages is dependent on p38. Human monocytes (A) and macrophages (B) were left unstimulated or stimulated with 20 μg/ml of Mf for 24 h with various concentrations of p38 inhibitor. Cytokines in the supernatants were detected by ELISA. Data are from 7–9 donors and shown as mean ±SEM. Statistical analysis was done using the Wilcoxon matched-pairs signed rank test. Values statistically different from the unstimulated control are depicted as ^§^ p<0.05 and ^§§^ p<0.01. Values statistically different from Mf-stimulated monocytes are depicted as * p<0.05 and **p<0.01.
(TIF)
