# Supplementary figures and images for: Differential immunomodulation in human monocytes versus macrophages by filarial cystatin
Source: PLoS One. 2017 Nov 15;12(11):e0188138. doi: 10.1371/journal.pone.0188138 (PMC5687743; doi:10.1371/journal.pone.0188138)

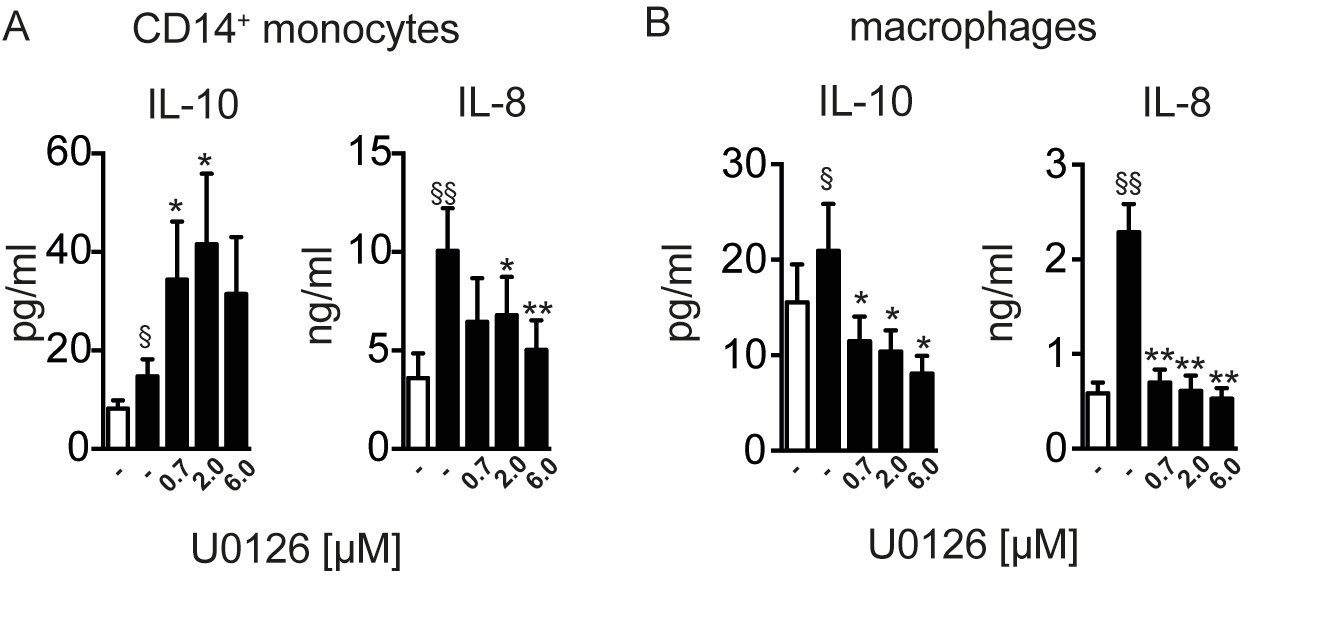

Supplement: S1 Fig — Human monocytes (A) and macrophages (B) were left unstimulated or stimulated with 20 μg/ml of Mf for 24 h with various concentrations of MEK1/2 inhibitor. Cytokines in the supernatants were detected by ELISA. Data are from 7–9 donors and shown as mean ±SEM. Statistical analysis was done using the Wilcoxon matched-pairs signed rank test. Values statistically different from the unstimulated control are depicted as § p<0.05 and §§ p<0.01. Values statistically different from Mf-stimulated monocytes are depicted as * p<0.05 and **p<0.01. (TIF) [file pone.0188138.s001.tif]

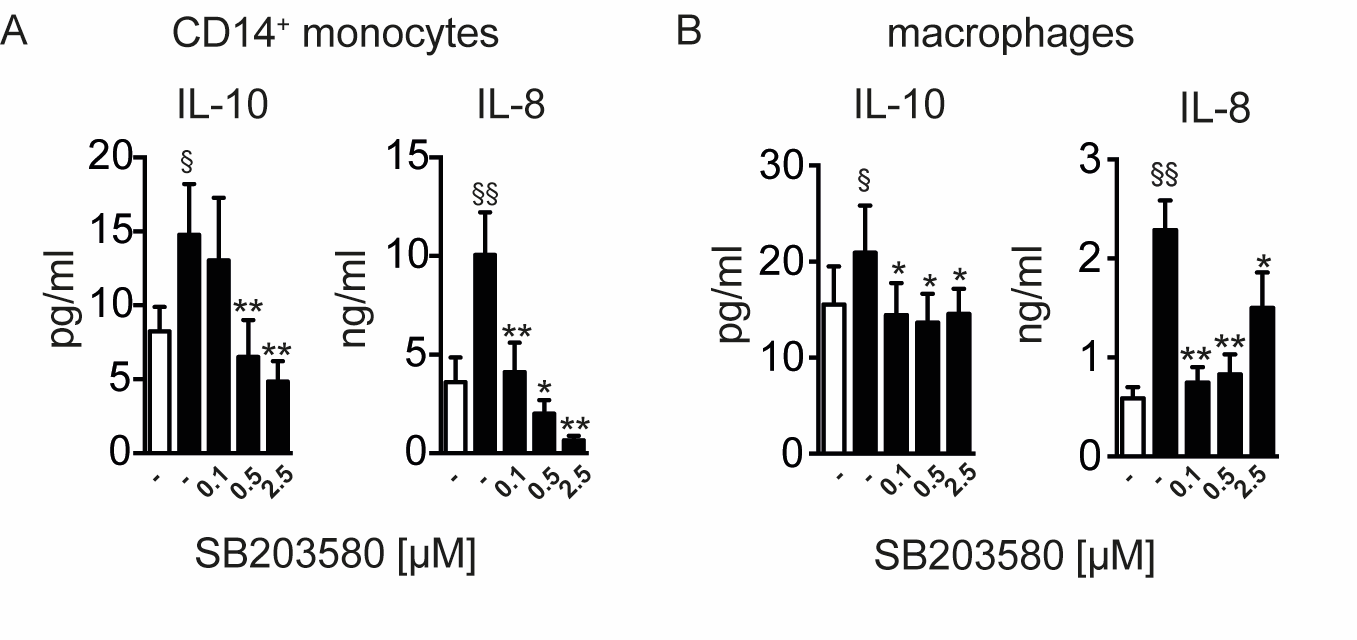

Supplement: S2 Fig — Human monocytes (A) and macrophages (B) were left unstimulated or stimulated with 20 μg/ml of Mf for 24 h with various concentrations of p38 inhibitor. Cytokines in the supernatants were detected by ELISA. Data are from 7–9 donors and shown as mean ±SEM. Statistical analysis was done using the Wilcoxon matched-pairs signed rank test. Values statistically different from the unstimulated control are depicted as § p<0.05 and §§ p<0.01. Values statistically different from Mf-stimulated monocytes are depicted as * p<0.05 and **p<0.01. (TIF) [file pone.0188138.s002.tif]
